# Supplementary material for: Portosystemic shunt placement reveals blood signatures for the development of hepatic encephalopathy through mass spectrometry
Source: Nat Commun. 2023 Aug 31;14:5303. doi: 10.1038/s41467-023-40741-9 (PMC10471626; doi:10.1038/s41467-023-40741-9)
Supplement: Supplementary file 1 — Supplementary Information [file 41467_2023_40741_MOESM1_ESM.pdf]

Portosystemic Shunt Placement Reveals Blood Signatures for the Development of Hepatic Encephalopathy through Mass Spectrometry

Ana Carolina Dantas Machado[1*], Stephany Flores Ramos[1,2,3*], Julia M. Gauglitz[4], Anne-Marie Fasslerr[5], Daniel Petras[4,6], Alexander A. Aksenov[4,7], Un Bi Kim[5], Michael Lazarowicz[8], Abbey Barnard Giustini[1,9,10], Hamed Aryafar[11,12], Irine Vodkin[1], Curtis Warren[5], Pieter C. Dorrestein[4,13,14,15], Ali Zarrinpar[5,16,17,#], Amir Zarrinpar[1,13,18,19,#]

********SUPPLEMENTARY MATERIAL********

Supplementary Figure 1. Flowchart of patient screening, consent, admission
Supplementary Figure 2. Feature-based molecular networking identifies metabolite features identified in plasma samples.
Supplementary Figure 3. Qurro feature ranking.
Supplementary Figure 4. Metabolome dissimilarities across participants based on HE grade.
Supplementary Figure 5. Levels of bile acids in plasma of post-TIPS participants based on HE grade.
Supplementary Figure 6. Chemical proportionality pre- to post-TIPS.
Supplementary Table 1. Demographic data of study participants

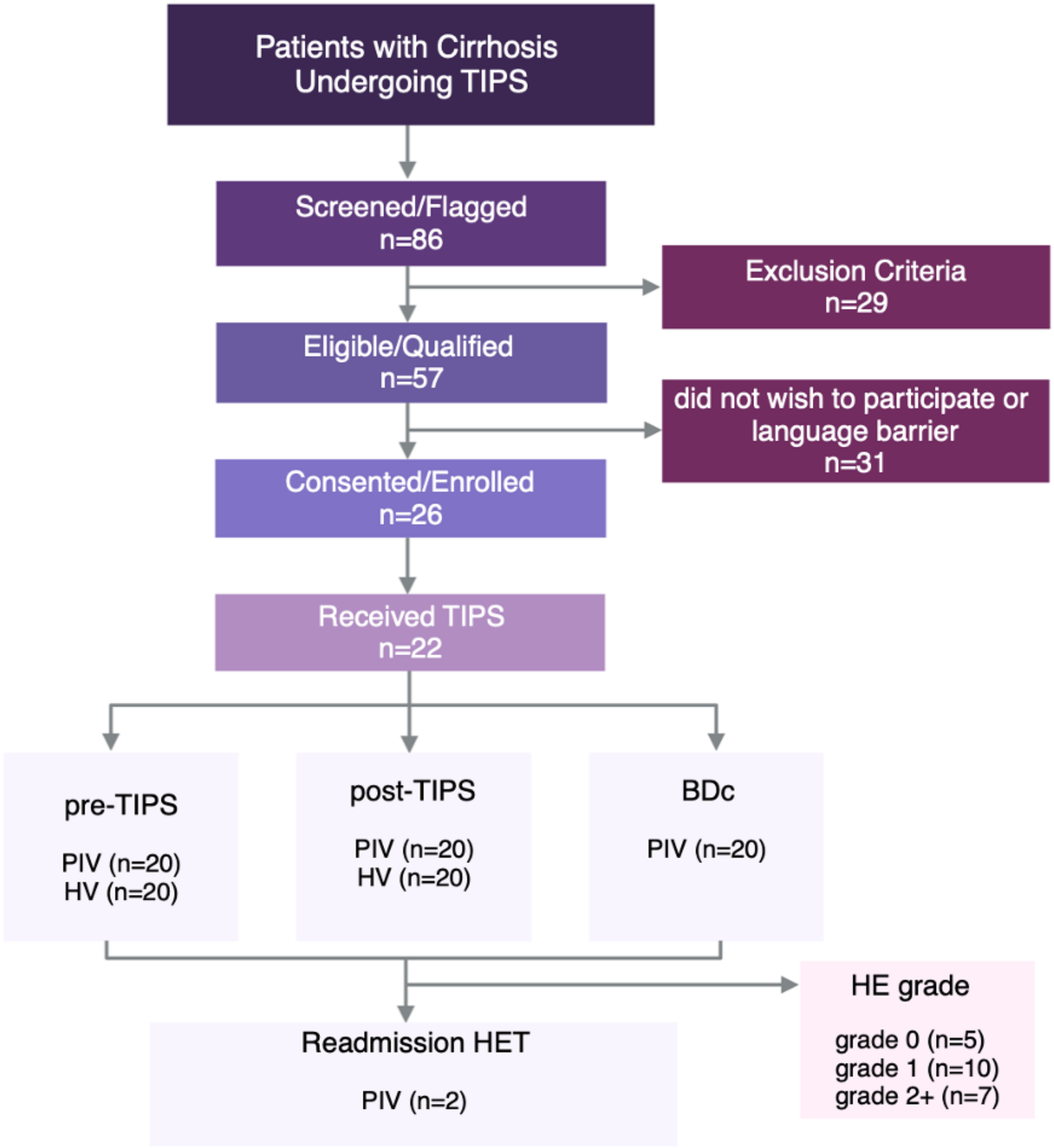

Supplementary Figure 1. Flowchart of patient screening, consent, admission. TIPS=transjugular intrahepatic portosystemic shunt, HE=hepatic encephalopathy, HET= hepatic encephalopathy treatment, PIV=peripheral vein, HV=hepatic vein. HE grade: 0=none; 1=mild; 2+=severe.

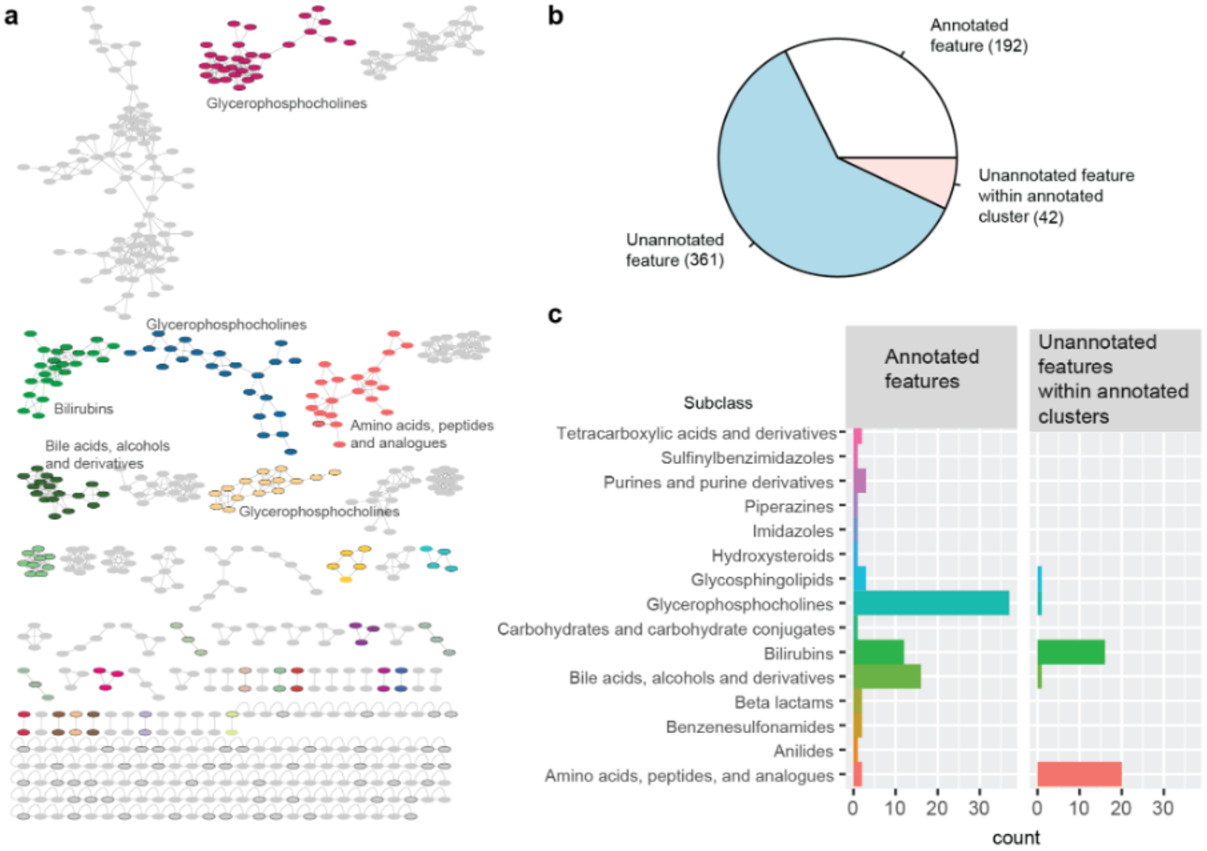

Supplementary Figure 2. Feature-based molecular networking identifies metabolite features identified in plasma samples. (**a**) Molecular network from all samples. Each node represents a feature or metabolite. Lines connect neighboring nodes. Colors represent different clusters with at least two nodes and one annotated feature. Subclass of main annotated clusters are shown. (**b**) Numbers of feature annotation: annotated features (n=192); features within annotated cluster (n=42); unannotated features (n=361). (**c**) Representation of metabolite subclass classification based on GNPS annotation.

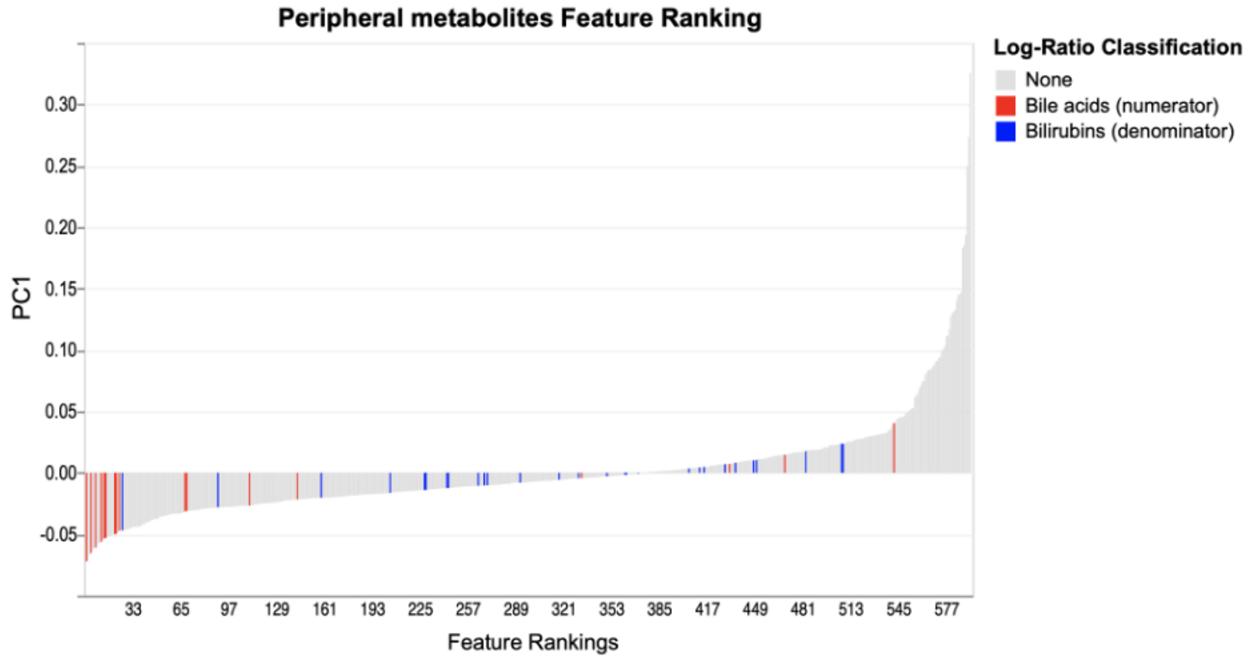

Supplementary Figure 3. Qurro feature ranking. The positioning of bile acids (red) vs. bilirubins (blue) in the peripheral first principal component (PC1) for the DEICODE-calculated β-diversity was used to calculate the natural log ratio.

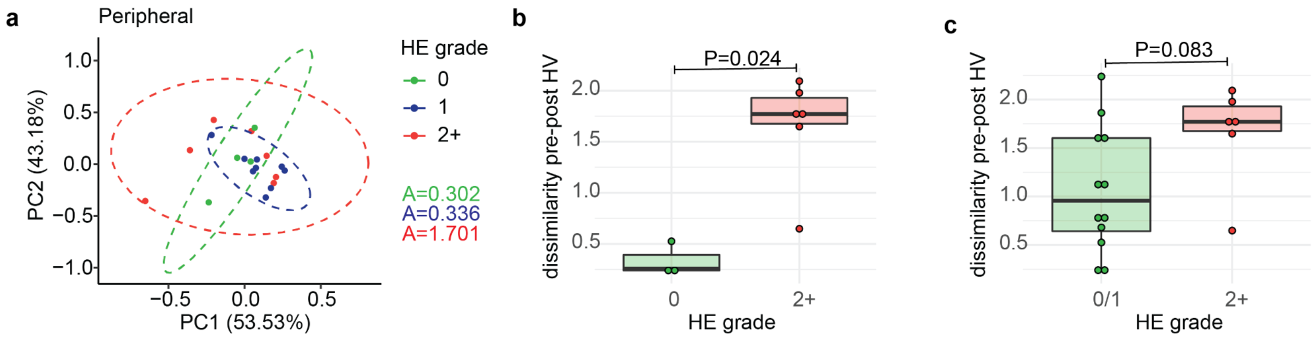

Supplementary Figure 4. Metabolome dissimilarities across participants based on HE grade. (**a**) RPCA of peripheral plasma metabolome dissimilarities based on HE grade. A is the area within the ellipsis. (**b-c**) Pre vs. Post TIPS comparison of hepatic vein metabolome dissimilarity between participants based on HE grade (Wilcoxon Test): (**b**) HE grades 0 (green, n=3) and 2+ (red, n=6); (**c**) HE grades 0/1 (green, n=12) and 2+ (red, n=6). The boxplots show median and upper and lower quartiles. The extreme lines show the highest and lowest value. The boxplot is overlaid with the visualization of single observations.

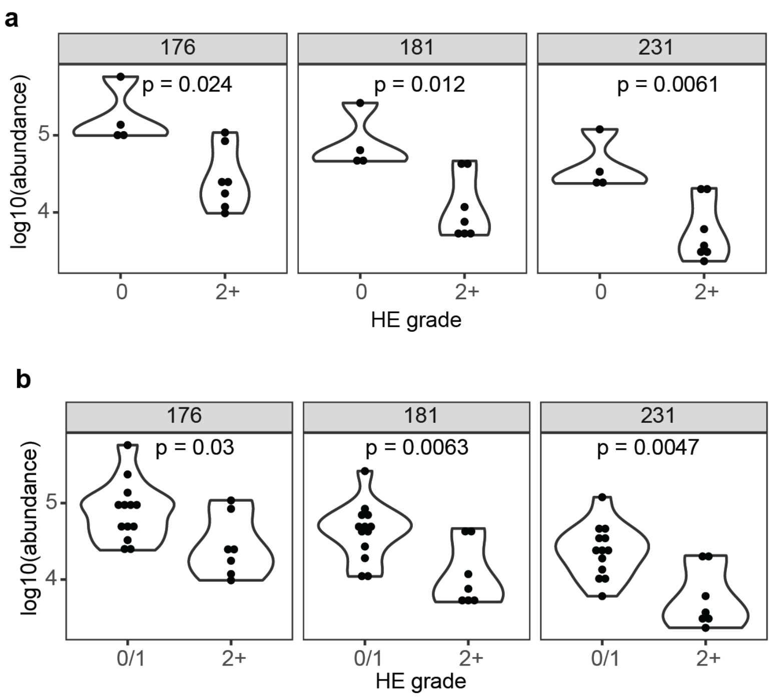

Supplementary Figure 5. **Levels of bile acids in plasma of post-TIPS participants based on HE grade.** (**a-b**) Bile acid levels and significant abundance differences in the post-TIPS peripheral blood based on HE grade: (**a**) HE grades 0 (n=4) and 2+ (n=7); (**b**) Bile acid levels and significant abundance differences between HE grades 0/1 (n=13) and 2+ (n=7). Significance was determined based on a two-sided Wilcoxon test.

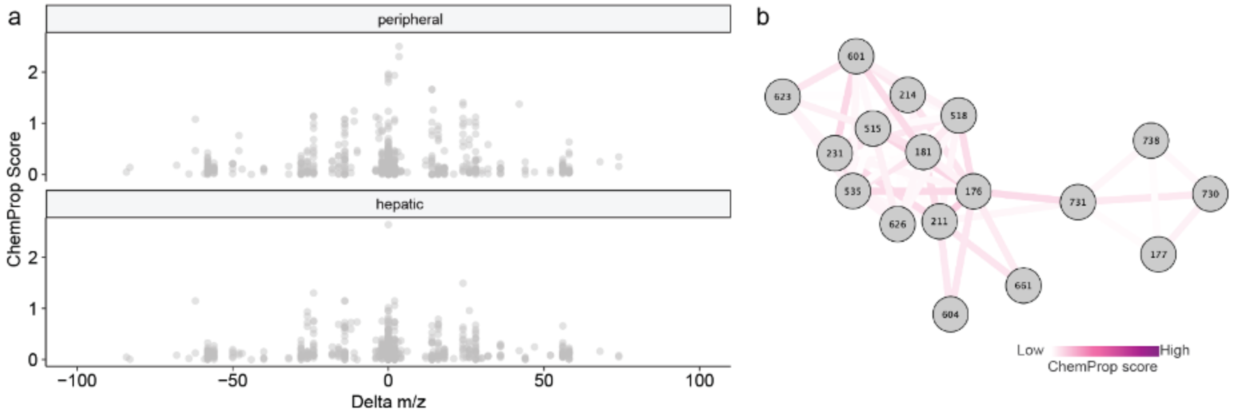

Supplementary Figure 6. Chemical proportionality pre- to post-TIPS. (**a**) ChemProp score and associated Delta m/z for each pair of neighboring metabolites. (**b**) Network representation of bile acids with associated IDs for individual metabolites and ChemProp scores for neighboring metabolite pairs.

Supplementary Table 1. Demographic data of study participants.

Demographic Variable	participant total (N=22)
**Female (%)**	8 (36%)
**Age (± SEM)**	59.8 ± 2.4
**Diagnosis* (%)**	
Alcohol	10 (45%)
Cryptogenic	3 (14%)
NASH	8 (36%)
HCV	2 (9%)
PBC	1 (5%)
**MELD (± SEM)**	12 ± 0.9
**Indications for TIPS* (%)**	
Ascites	20 (91%)
Esophageal Varices	18 (82%)
Rectal Varices	2 (9%)
**Post-TIPS HE (%)**	17 (77%)
**Post-TIPS HE Grade (%)**	
0	5 (23%)
1	10 (45%)
2+	7 (32%)

*some patients had more than one diagnosis or indication for TIPS
NASH= nonalcoholic steatohepatitis; HCV= hepatitis C virus; PBC= primary biliary cirrhosis; MELD=Model for end stage liver disease; TIPS= transjugular intrahepatic portosystemic shunt; HE= hepatic encephalopathy. HE grade scored based on West Haven criteria. There is no discordance between self-reported sex and gender in our patient cohort.
