# Supplementary material for: Portosystemic shunt placement reveals blood signatures for the development of hepatic encephalopathy through mass spectrometry
Source: Nat Commun. 2023 Aug 31;14:5303. doi: 10.1038/s41467-023-40741-9 (PMC10471626; doi:10.1038/s41467-023-40741-9)
Supplement: Supplementary file 3 — Reporting Summary [file 41467_2023_40741_MOESM3_ESM.pdf]

nature portfolio

Corresponding author(s): Amir Zarrinpar, MD, PhD

Last updated by author(s): Jul 19, 2023

Reporting Summary

Nature Portfolio wishes to improve the reproducibility of the work that we publish. This form provides structure for consistency and transparency in reporting. For further information on Nature Portfolio policies, see our Editorial Policies and the Editorial Policy Checklist.

Statistics

For all statistical analyses, confirm that the following items are present in the figure legend, table legend, main text, or Methods section.

n/a	Confirmed	
☐	☒	The exact sample size (*n*) for each experimental group/condition, given as a discrete number and unit of measurement
☐	☒	A statement on whether measurements were taken from distinct samples or whether the same sample was measured repeatedly
☐	☒	The statistical test(s) used AND whether they are one- or two-sided<br>*Only common tests should be described solely by name; describe more complex techniques in the Methods section.*
☐	☒	A description of all covariates tested
☐	☒	A description of any assumptions or corrections, such as tests of normality and adjustment for multiple comparisons
☐	☒	A full description of the statistical parameters including central tendency (e.g. means) or other basic estimates (e.g. regression coefficient) AND variation (e.g. standard deviation) or associated estimates of uncertainty (e.g. confidence intervals)
☐	☒	For null hypothesis testing, the test statistic (e.g. *F*, *t*, *r*) with confidence intervals, effect sizes, degrees of freedom and *P* value noted<br>*Give P values as exact values whenever suitable.*
☒	☐	For Bayesian analysis, information on the choice of priors and Markov chain Monte Carlo settings
☒	☐	For hierarchical and complex designs, identification of the appropriate level for tests and full reporting of outcomes
☒	☐	Estimates of effect sizes (e.g. Cohen's *d*, Pearson's *r*), indicating how they were calculated

Our web collection on statistics for biologists contains articles on many of the points above.

Software and code

Policy information about availability of computer code

Data collection	REDcap 13.8.1 - © 2023 Vanderbilt University; Microsoft Excel Version 16.75
Data analysis	R version 4.1.0; Qiime2 version 2021.4. Code to perform analysis of metabolomics data is available on GitHub [https://github.com/acdm2020/HE-TIPS/releases/tag/v1.0] and Zenodo [DOI: 10.5281/zenodo.8206272]

For manuscripts utilizing custom algorithms or software that are central to the research but not yet described in published literature, software must be made available to editors and reviewers. We strongly encourage code deposition in a community repository (e.g. GitHub). See the Nature Portfolio guidelines for submitting code & software for further information.

Data

Policy information about availability of data

All manuscripts must include a data availability statement. This statement should provide the following information, where applicable:

- Accession codes, unique identifiers, or web links for publicly available datasets
- A description of any restrictions on data availability
- For clinical datasets or third party data, please ensure that the statement adheres to our policy

Raw and processed mass spectrometry data is available on MassIVE under the MassIVE ID MSV000090443.

Research involving human participants, their data, or biological material

Policy information about studies with human participants or human data. See also policy information about sex, gender (identity/presentation), and sexual orientation and race, ethnicity and racism.

Reporting on sex and gender	Table S1 in our manuscripts reports patient sex. Sex and gender were self-reported. There is no discordance between sex and gender in our patient cohort. This has now been clarified in Table S1.
Reporting on race, ethnicity, or other socially relevant groupings	NIH-defined race and ethnicity information was provided to the participants. The participants self-reported which ethnicity or race with which they identified. No confounds of race or ethnicity were found in the analysis of the data.
Population characteristics	Data of study participants: Participant total (n = 22); Female = 36%; Age (+/- SEM) = 59.8 ± 2.4; Diagnosis: alcohol (45%), cryptogenic (14%), NASH (36%), HCV (9%), PBC (5%); MELD (± SEM) = 12 ± 0.9; Indications for TIPS: Ascites (91%), Esophageal Varices (82%), Rectal Varices (9%); Post-TIPS HE = 77%; Post-TIPS HE Grade: grade 0 (23%), grade 1 (45%), grade 2+ (32%).
Recruitment	Subjects undergoing elective TIPS procedure were recruited from the Hepatology clinic, Interventional Radiology clinic and EPIC search. A partial waiver of HIPPA was required so that the study coordinator could review existing medical records in EPIC and the clinics, identify patients who may meet inclusion criteria. Study coordinators identified subjects through clinic providers who agreed to the pre-screened and were invited to participate by their primary care provider. Only study staff would recruit potential participants and the participant's doctor would not recruit. Patients that were interested in participating were given further information with the study coordinator's name and phone number to contact. Participants did not receive compensation for participation in the study.
Ethics oversight	This is a multi-center prospective cohort study approved by the IRBs of the UC San Diego and University of Florida

Note that full information on the approval of the study protocol must also be provided in the manuscript.

Field-specific reporting

Please select the one below that is the best fit for your research. If you are not sure, read the appropriate sections before making your selection.

- [x] Life sciences
- [ ] Behavioural & social sciences
- [ ] Ecological, evolutionary & environmental sciences

For a reference copy of the document with all sections, see nature.com/documents/nr-reporting-summary-flat.pdf

Life sciences study design

All studies must disclose on these points even when the disclosure is negative.

Sample size	Power analysis during study design was performed based on the assumption that BA levels increase ten-fold in patients with cirrhosis compared to healthy controls (46.0 uM). To determine the number of participants needed, we assumed that the average patient with cirrhosis has a mean total BA level that is 10-fold higher than normal (46.0 #M). To be able to detect a difference of 30% change in total BA pool, assuming a within group standard deviation of 11.0% (based on standard deviation of total BAs in populations of cirrhotic patients) with an ! = 0.05 and power (1-") = 0.8 (power analysis for paired t-test analysis), we would need a minimum of 5 participants. Since we wanted to have samples from at least 5 participants while they are admitted to the hospital for overt HE, we recruited more participants into the study. The likelihood of a patient post-TIPS being admitted for overt HE is 35% though it can vary by facility. If we estimate that admission for HE after TIPS at our facility is 25%, we will need to recruit 20 participants to be able to attain 5 samples from patients hospitalized for overt HE. We also performed a retrospective power analysis that showed our sample sizes are sufficient to find a difference of 82% between HE grade 0 and the other groups (1-beta = 80%; alpha 5%). The fact that our actual differences between HE grade 0 and 1 is 391% and HE grade 0 and 2+ is 492% demonstrates that we have more than enough power in our sample size to detect differences in our groups.
Data exclusions	As presented in Table S1, 86 patients were screened, of which 29 met the exclusion criteria described in our methods. of the 57 participants who were eligible, 31 chose to not participate or had a language barrier. Thus 26 patients were consented for the study. Of these 26, 4 did not receive TIPS during their procedure, and we collected specimens from the 22 patients who underwent the procedure.
Replication	Replication is not applicable as this was a clinical study of a single intervention in each patient that cannot be replicated in an individual.
Randomization	Randomization is not applicable as this was an observational clinical study, not an intervention trial.
Blinding	Those performing metabolomics analysis were blinded to samples that were being run.

Reporting for specific materials, systems and methods

We require information from authors about some types of materials, experimental systems and methods used in many studies. Here, indicate whether each material, system or method listed is relevant to your study. If you are not sure if a list item applies to your research, read the appropriate section before selecting a response.

Materials & experimental systems

n/a	Involved in the study
[x]	[ ] Antibodies
[x]	[ ] Eukaryotic cell lines
[x]	[ ] Palaeontology and archaeology
[x]	[ ] Animals and other organisms
[x]	[ ] Clinical data
[x]	[ ] Dual use research of concern
[x]	[ ] Plants

Methods

n/a	Involved in the study
[x]	[ ] ChIP-seq
[x]	[ ] Flow cytometry
[x]	[ ] MRI-based neuroimaging
